# Feeding Practices and Undernutrition in 6–23-Month-Old Children of Orthodox Christian Mothers in Rural Tigray, Ethiopia: Longitudinal Study

**DOI:** 10.3390/nu11010138

**Published:** 2019-01-10

**Authors:** Beruk Berhanu Desalegn, Christine Lambert, Simon Riedel, Tegene Negese, Hans Konrad Biesalski

**Affiliations:** 1 College of Agriculture, Hawassa University, Hawassa 05, Ethiopia; tegenengss38@gmail.com; 2 Institute of Biological Chemistry and Nutrition, University of Hohenheim, Garbenstr. 30, 70593 Stuttgart, Germany; christine.lambert@uni-hohenheim.de (C.L.); simon.riedel@uni-hohenheim.de (S.R.); biesal@uni-hohenheim.de (H.K.B.)

**Keywords:** stunting, wasting, undernutrition, minimum acceptable diet, minimum diet diversity, Tigray, Orthodox fasting, Ethiopia

## Abstract

Fasting period and fasting status affect the feeding practices and nutritional status of Ethiopian Orthodox mothers. Even if children are exempted from fasting, some mothers do not prepare their food from animal sources as it could contaminate utensils for cooking family foods. Therefore, the objective of this study was to assess feeding practices and undernutrition in 6–23-months old children whose mothers are Ethiopian Orthodox religion followers during lent fasting and non-fasting periods in rural Tigray, Northern Ethiopia, and to identify associated factors. A community-based longitudinal study was carried out in Ethiopian Orthodox lent fasting and non-fasting periods. Using a multi-stage systematic random sampling technique, 567 and 522 children aged 6–23 months old participated in the fasting and non-fasting assessments, respectively. Statistical analyses were done using logistic regression, an independent sample *t*-test, Wilcoxon signed-rank (WSRT) and McNemar’s tests. The prevalences of stunting, underweight and wasting were 31.6–33.7%, 11.7–15.7% and 4.4–4.8%, respectively. The weight-for-height (WHZ) and height-for-age (HAZ) values for children of fasting mothers were significantly lower (*p* < 0.05) compared to those of non-fasting mothers. Likewise, the median weight-for-age (WAZ) and diet diversity score (DDS) of children of fasting mothers were also significantly higher in non-fasting than in fasting periods. A small proportion of children (2.3–6.7%) met the minimum acceptable diet (MAD) in the study population, but these measures were significantly increased (*p* < 0.001) in the children of non-fasting mothers. Mother’s fasting during lactation period of the indexed child was amongst the independent factors common in child stunting, underweight and wasting. Nutritional status and feeding practices of 6–23-month-old children are affected by maternal fasting during the fasting period. Therefore, without involvement of religious institutions in the existing nutritional activities, reduction of undernutrition would not be successful and sustainable.

## 1. Introduction

Despite the efforts undertaken globally, little has been achieved in reducing undernutrition with a sizeable gap from the global targets and goals still to fill. Worldwide, the number of stunted and wasted children under five years of age were about 154.8 and 52 million, respectively, in 2016, while more than 40 million children are overweight [1]. About 38%, 27% and 24% of stunted, wasted and overweight children under five years of age, respectively, were from Africa. Even worse, the number of stunted and overweight children increased by 9 and 9.8 million, respectively, between 2000 and 2016 [1]. The long-term impact of malnutrition on people’s lives, most notably in health, education, and productivity, highly affects the human capital of a country. For example, an estimated loss of 4.7 billion US$ (equivalent to 16.5% of the national GDP) was recorded in 2009 due to child undernutrition in Ethiopia [2]. Therefore, the government of Ethiopia has been following different approaches to reduce maternal and childhood undernutrition significantly. Recently, the “Seqota” Declaration (2015–2030) was launched with the goal of eliminating all forms of malnutrition among children under age 2 by 2030 [3]. However, this cannot be achieved unless the socio-cultural and religious issues related with feeding practices are deeply investigated and addressed in Ethiopia, where more than 85 ethnic groups with diversified religious practices exist. As these practices have been passed from one generation to the next generation, they might have resulted in an intergenerational cycle of malnutrition. This could be worse in physiologically nutrient needy groups like pregnant and lactating mothers, and young and growing children.

Previous studies in Ethiopia and other parts of the world showed that maternal nutritional status and feeding practices are associated with growth indices of their children [4,5,6,7,8]. A more recent study by Desalegn and colleagues showed that maternal Body Mass Index (BMI) and dietary pattern of lactating mothers were negatively affected by maternal fasting status, and during Ethiopian Orthodox lent fasting period regardless of fasting status in rural Ethiopia [9]. Even if children are exempted from fasting, some mothers said that they were not happy to prepare food for them from animal sources as it could contaminate utensils for cooking family food [10]. Furthermore, a study on 6–23-month-old children of Ethiopian Orthodox mothers from the Gojam district also showed that mothers/caregivers who did not feed a diet containing animal products to their children due to fear of utensil contamination for family food preparation were less likely to feed the recommended dietary diversity. Economic limitations were a minor reason [11]. However, to the best of our knowledge, the effect of maternal fasting status and feeding practices on the nutritional status of the children and how these differ between fasting and non-fasting periods are not known. Therefore, the purpose of this study was to assess and compare the feeding practices and nutritional status of 6–23-month-old children of fasting and non-fasting lactating mothers during Ethiopian Orthodox lent fasting and non-fasting periods in rural Tigray of Ethiopia, where the majority are Orthodox religion followers.

## 2. Materials and Methods

### 2.1. Study Area, Design, Participants and Sampling

A longitudinal community-based survey was conducted in lent fasting (15 February–15 April 2017) and non-fasting (1–30 May 2017) periods in rural Genta Afeshum woreda (the third-level administrative division in Ethiopia), in rural Tigray, Ethiopia. The district is one of the hot spot areas of food insecurity and has a total population of 99,112 and 19 health posts to serve the community. Almost all people in the woreda belong to the ethnic group of Tigray and are followers of Ethiopian Orthodox Christianity.

For this study, the sample size was calculated using a single population proportion formula and considering the prevalence of stunting (57.1%) elsewhere in Tigray region [12], 95% of confidence interval for true prevalence and a relative precision (d) of 5%. The total population of children aged between 6–23 months old in the district was 4906, so that the finite source population size correction formula was used. Additionally, a 1.5 design effect and 10% non-response rate was used in the calculated sample size so as to get the final sample size of 575.

To obtain representative samples, multi-stage systematic random sampling was used. First, of the three woredas where the Deutsche Gesellschaft für Internationale Zusammenarbeit (GIZ, Bonn, Germany) are implementing a nutrition sensitive agriculture (NSA) project in Ethiopia, Genta Afeshum was randomly selected. Then, seven kebeles (smallest administrative unit in Ethiopia) were selected randomly out of the twenty kebeles residing in the woreda. Children who were aged between 6–23 months, apparently healthy and breastfeeding during the study periods, were included, whereas children who were twins and whose mothers were not permanent residents of the study area during the study period were excluded from the study. Based on these, the list of children was prepared by the health extension workers in the selected kebeles, and finally the samples were selected using systematic random sampling methods.

### 2.2. Data Collection

Trained and experienced data collectors who are fluent in Tigrigna, Amharic and English languages were recruited. First, the questionnaire was prepared by the principal investigator, considering the information needed for the study, translated to the local language Tigrigna by a professional translator and pre-tested for its appropriateness. Using this, information on socio-demographic and economic characteristics, maternal and child characteristics, water, sanitation and health (WASH) and feeding practices was collected. The household food insecurity information was also collected using a household food insecurity access scale (HFIAS) standard questionnaire developed by Food and Nutrition Technical Assistance Project (FANTA) [13].

The weight of children was measured using a calibrated portable digital scale (Seca 770, Hanover, Germany) working with powered battery and measured to the nearest 0.1 kg. Likewise, the length of children was measured in a lying position with a wooden board to the nearest 0.1 cm. During weight and length measurements, the mothers were advised to remove their child’s clothes and shoes until the child had only light clothes to minimize the weight and to get actual length, respectively. Duplicate measurements (length and weight) were carried out following standard procedures. Minimum diet diversity (MDD), minimum meal frequency (MMF) and minimum acceptable diet (MAD) of the children were computed from the two 24-h recall data points collected preceding the survey.

Ethical approval was obtained from the institutional review board of the College of Health Sciences at Hawassa University and Tigray region Health Bureau in Ethiopia and Ethics Commission, Landesärztekammer Baden-Württemberg, Germany. Permission was also obtained from the Genta Afeshum woreda Health Office in the Tigray Province of Ethiopia. Additionally, the purpose of the study and the confidentiality of the information to be collected were explained to the mothers, and their agreement to participate with their indexed child in the study was documented by signing the informed consent. Each participating mother was also told that whenever the mothers felt they wanted to discontinue the participation with their child, withdrawal from the study was possible.

### 2.3. Data Management and Analysis

Before submitting the data, variable coding was conducted in SPSS for window version 20 (IBM Corporation, Armonk, NY, USA). Following this, the data were entered, cleaned and analyzed. First, frequency and crosstab were conducted to check completeness of data and to present the results in descriptive statistics (frequency and percent).

In our case, the diet diversity score was calculated as the summation of the number of food groups consumed for each of the two days, and averaged. Seven food groups were created, which included: grains, roots and tubers; legumes and nuts; dairy products; flesh foods; eggs; vitamin A-rich fruits and vegetables; and other fruits and vegetables. Likewise, meal frequency was computed as sum of the number of solid, semi-solid, or soft foods eaten by the child within 24-h for each of the two days, and averaged. A child who ate an average of at least four food groups in their meals of the two days fulfilled MDD. In breastfed children, the MMF was two times if aged 6–8 months and three times for 9–23 months. If a child fulfilled both the criteria for MDD and MMF, then he/she was considered as having met MAD.

The nutritional status (stunting, underweight and wasting) was identified using length-for-age, weight-for-age, and weight-for-height Z scores and compared with WHO reference data. For this, the height, weight, age and sex data of the children were entered in WHO Anthro software version 3.2.2 (World Health Organization, Geneva, Switzerland). A child who was below -2 standard deviations (-2SD) for HAZ, WHZ or WAZ was considered as stunted, wasted or underweight, respectively.

First, potential predicting variables for the outcome variables (stunting, underweight and wasting) were assessed from previous studies and included in bivariate analysis, and those with *p*-value < 0.25 were selected for multivariate logistic regression. Then, multi-collinearity was checked between the candidate variables separately for the three outcome variables using a variance inflation factor (VIF), and all were less than 10. Then, the candidate variables were entered in a multivariate logistic regression model with the stepwise forward Wald method. A *p*-value < 0.05 was used to declare the variables as predictors of the outcome variables. Hosmer and Lemeshow test and C-statistics (Area under the curve (AUC)) were used for model tests and were verified. Normality of continuous data was checked using the Kolmogorov-Smirnov test. An independent sample *t*-test was used to identify the difference between the children of fasting and non-fasting mothers’ WAZ, WHZ and WAZ score values during the lent fasting period. Non-normally distributed data were analyzed using the Wilcoxon signed-rank test, whereas dichotomous data were analyzed by McNemar’s test to identify the difference between children of fasting and non-fasting mothers’ sub-groups dietary patterns between fasting and non-fasting periods.

## 3. Results

A survey was conducted with 567 children during Ethiopian Orthodox lent fasting with a response rate of 98.6%. Of those children who participated in the lent fasting survey, 505 were again involved in the non-fasting period, with a response rate of 87.8%. The reasons for excluding some children of the non-fasting period for the follow-up study were that some were older than 23 months or migrated with their family to other areas or were absent during the 2nd round survey.

### 3.1. Socio-Demographic Characteristics

In this study, almost all children belong to mothers who were from Ethiopian Orthodox Christians. Most of the mothers were house-wives, and more than half of the mothers were literate. About one-third of the mothers included in the study were fasting while they were pregnant and lactating. Only about one-third of 6–23-month-old children were from food secured and small family size households (HH) although most of them were from HH that owned toilets (Table 1).

### 3.2. Nutritional Status and Consumption Pattern

Almost all children included in this study consumed colostrum during the first few days after birth. More children were stunted (31.6–33.7%) than underweight (11.7–15.7%) and wasted (4.4–4.8%) in the study. Consumption of grains, roots and tubers, legumes and nuts, and fruits and vegetable foods was lower in children born of fasting than non-fasting mothers, both in fasting and non-fasting periods. Likewise, the proportion of 6–23-month-old children of non-fasting mothers who consumed dairy products, and food groups containing egg was higher compared to children of fasting mothers, both in fasting and non-fasting periods. Additionally, the proportion of 6–23-month-old children who consumed meat and vitamin A-rich fruits and vegetables was higher during non-fasting than fasting periods. Moreover, a lower proportion of 6–23-month-old children fulfilled the MMF, MDD and MAD standards during fasting (74.3%, 2.5% and 2.3%) than during non-fasting periods (75.0%, 6.9% and 6.7%), respectively (Table 2).

### 3.3. Factors Associated with Child Undernutrition

Children between 13–23 months of age were about 1.8 times more likely to be underweight than those who were between 6–12 months (AOR = 1.79 (1.07, 3.0)). Children who were born of mothers fasting during their pregnancy and lactation periods had 2.1 and 2.6 times higher odds of being underweight than those born of mothers not fasting during their pregnancy and lactation periods (AOR = 2.12 (1.29, 3.51) and AOR = 2.57 (1.56, 4.23), respectively). Children from illiterate and farming mothers were 1.8 and 2.3 times more likely to be underweight compared to those whose mothers were literate and housewives (AOR = 1.79 (1.10, 2.90) and AOR = 2.28 (1.25, 4.16), respectively). Similarly, children between 13–23 months of age had 3.1 times higher odds of stunting than those between 6–12 months of age (AOR = 3.07 (2.04, 4.63)).

Children who did not consume colostrum after their birth were about 4.1 times as likely to be stunted compared to those who did (AOR = 4.10 (1.42, 11.82)). Additionally, children born of mothers fasting during their pregnancy and lactation periods were 1.8 times more likely to be stunted (AOR = 1.83 (1.21, 2.77)) than those children from mothers who did not fast during both periods (AOR = 1.81 (1.20, 2.72)). Furthermore, children living in households who did not own a latrine were 1.7 times more likely to be stunted compared to those owning a latrine (AOR = 1.67 (1.06, 2.64)). Children whose mothers were fasting during their lactation period and who were illiterate were 6.8 and 2.2 times more exposed to wasting when compared with the references (not fasting and literate, respectively) (Table 3).

### 3.4. Trend of Food Consumption Pattern and Nutritional Status of 6–23-Month-Old Children

Based on McNemar’s test, there was no significant difference (*p* > 0.05) in the consumption of all of the seven food groups during the lent fasting period between children born of fasting and non-fasting mothers. Likewise, the proportion of children of fasting and non-fasting mothers’ sub-groups who fulfilled the MMF, MDD, and MAD criteria during the lent fasting period was not significantly different (*p* > 0.05). However, the proportion of children of non-fasting mothers who consumed grains, roots and tubers, pulses and nuts, other fruits and vegetables, and egg food groups was significantly higher than the fasting mothers’ sub-group (*p* ≤ 0.001, *p* = 0.047, *p* ≤ 0.001 and *p* = 0.017, respectively). Similarly, the proportion of children who fulfilled the MDD and MAD significantly increased in the non-fasting mothers sub-group compared to the fasting mothers sub-group during the non-fasting period (*p* ≤ 0.001) (Table 4).

Likewise, the WAZ, WHZ and HAZ of children of non-fasting mothers were significantly higher than those of children of the fasting sub-group during the lent fasting period (*p* ≤ 0.001, *p* ≤ 0.001 and *p* = 0.003, respectively) (Table 5). A Wilcoxon signed-rank test showed that except for the median of WAZ (*p* = 0.153) for children of the non-fasting mothers’ sub-group, the median WAZ (*p* = 0.034) and DDS were significantly increased in the non-fasting than in the fasting period in children from both fasting and non-fasting mothers’ sub-groups (*p* ≤ 0.001) (Table 6).

## 4. Discussion

This is the first time a study has assessed feeding practices and nutritional status of 6–23-month-old children of fasting and non-fasting mothers during the Ethiopian Orthodox lent fasting and non-fasting periods in rural Tigray, Ethiopia. Socio-economic factors, which are possibly associated with stunting, underweight and wasting, were also determined.

### 4.1. Food Consumption Pattern of 6–23-Month-Old Children

According to the UNICEF conceptual framework of malnutrition, inadequate dietary intake is one of the immediate causes of child malnutrition [14]. In our study, cereals, roots and tubers, and pulses and nuts are the food groups majorly prepared for the 6–23-month-old children in the rural Tigray in Ethiopia, regardless of fasting period. This result is consistent with previous studies in Ethiopia [11,15,16,17,18,19,20]. A similar finding was reported on the consumption of grains, roots and tubers for children from Vietnam, Bangladesh, Pakistan, Indian and the U.S. [21,22,23,24,25]. According to Demissie and his colleagues [21], consumption of fruits and vegetables is sub-optimal in the Tigray region and Ethiopia in general [26]. Supporting this evidence, we also found that only 1% of children consumed vitamin A-rich fruits and vegetables. The result is also comparable with a previous study in rural Tigray [27]. However, our finding was lower than findings of two national surveys (14.8, 27.7%) in Ethiopia and Pakistan (18.7%) [22,28,29]. This could be related to the fact that cultivation on the farm and availability in the markets are rare in the study area, probably resulting in a less diverse diet. Another explanation could be a difference in feeding practices [26]. However, the consumption of other fruits and vegetables food group was between 27.0–37.6% in the present study. This result is higher than national figures, which are 3.3% and 10.1% [28,29].

Animal source foods are both energy and nutrient dense, and an excellent source of high quality and easily digestible protein, in addition to being an efficient and easily absorbable source of micronutrients like iron, zinc, vitamin A, vitamin B12, riboflavin and calcium [30]. In the present study, the consumption of a diet composed of flesh foods was 0.5% and 1.6% in fasting and non-fasting periods, respectively. These results are comparable with a previous study during the Ethiopian Orthodox fasting period in the Dejen district (0.0%), and studies conducted in Gobalafto district in North Wollo (0–5%), Wolaita district (1.6%), and findings of the Ethiopian national food consumption survey for all regions in the country (0.1–0.6%) [11,15,31,32]. However, our finding was lower than a study conducted in the Arsi-Negele district of Oromia region, in Ethiopia (11.3%) and Mongolia (4–14%) [33,34]. This might be due to the fact that the area of the later studies has animal husbandry potential and cultural and eating habit differences. In the current study, consumption of dairy products was between 8.5% and 9.9% in fasting and non-fasting periods, respectively. An almost comparable proportion of 6–23-month-old children (7.5%) of the Dejen district consumed dairy products in the fasting period in North-west Ethiopia [11]. However, the findings in the present study were lower than in studies conducted in Amhara (about 20.0%) and Oromia (52.4%) regions, and Addis Ababa City (70.4%) in Ethiopia and Pakistan (50.5%) [17,20,22,34]. An explanation for this could be that the studies were conducted in different periods, and dairy products were the second most common foods groups consumed by the Pakistan children. In the present study, the proportions of children who consumed egg in the days before the surveys of fasting and non-fasting periods were 18.5% and 24.8%. However, these were lower than in similar studies in the Amhara region, which was 98.0%, where fasting of Ethiopian Orthodox religion is routine in the fasting days of the year. This could be due to more chickens in the households included in the later study, which may result in availability of eggs for children during the fasting period and household consumption in general. The consumption of egg in our finding was higher than the national findings of the last two Ethiopian demographic and health survey (DHS) studies (8.3%, 17%) and study in Amhara region (<10.0%) in Ethiopia [15,28,29]. However, it was consistent with a study conducted in the Arsi-Negele district (22.3%) of Oromia region, in Ethiopia [34].

Minimum meal frequency is a proxy indicator to determine whether the energy requirement of a child is met and examines the frequency of meals where the children received foods other than breastmilk, considering specific age and breastfeeding status of the child [29]. In this study, three-fourths of children of fasting and non-fasting mothers had eaten the minimum number of meals in the days preceding the fasting and non-fasting surveys. This result is in line with studies conducted in the Dabat (69.3–75.0%) and Gorche (78.0%) districts of northern and southern Ethiopia, respectively [35,36]. However, our finding is higher than studies in Kemba (14.5–33.6%), Bale (44.7%), Arsi-Negele (67.3%), Wolaita (69.3%) districts, elsewhere in Tigray region (66.0%) and findings for the Tigray region (55.6%, 49.4%), and national averages (47.9%, 44.6%) in Ethiopian DHS studies of 2011 and 2016, respectively, and Pakistan (38.2%) [16,19,28,29,34,37,38]. However, it is lower than findings on children from the South Wollo district, in Ethiopia, which were 91.0–97.0% and Nepal (87.8–98.2%) [15,39].

Minimum dietary diversity is one of the indicators to assess the quality of food consumed by 6–23-month-old children. Consumption of at least four different food groups in a day is the minimum requirement [29,40]. In this study, the proportion of children who consumed foods prepared from at least four diversified food groups (MDD) was 2.5% and 6.9% in fasting and non-fasting periods, respectively. This finding indicates that children aged 6–23 months in the study area are consuming a poor quality complementary diet. A study conducted in 6–23-month- old children of Orthodox Christian mothers in Gojam indicated that 13.6% had four diversified food groups in the food they consumed preceding the survey during the fasting period [11]. Similarly, our findings are also lower than findings in children living in the Gorche (10.4%), Kemba (23.3%), Bale (28.5%), and Wollo (23.0–41.0%) districts in Ethiopia, India (38%), Bangladesh (48.4%) and Vietnam (83.2%) [15,16,25,35,41,42]. Generally, these findings indicate that undernutrition specifically stunting and micronutrient deficiencies could be the public health problem in the area. This is supported by studies conducted in children from different parts of the world and ours [43,44,45,46].

For appropriate growth and development, infants and young children should be fed with at least four diversified food groups from the minimum number of meals (MAD) recommended according to specific to the age and breastfeeding status of the child [40]. In the present study, the proportion of 6–23-month-old children who met both the minimum diversity and the number of meals (MAD) preceding the surveys was 2.3–6.7% in the study population. The result is consistent with DHS 2011 and 2016 findings for the Tigray region (4.5% and 5.7%, respectively) and the national figure (4.0%) in DHS 2016 for Ethiopia [28,29]. However, it is lower than research findings conducted in three districts (17.0%) of the Tigray region, in northern Ethiopia, India (9%), Nepal (32%), Bangladesh (40%) and Sri Lanka (68%) [31,38,47]. Moreover, the proportion of 6–23-month-old children who fulfilled the criteria for MAD was higher for non-fasting mothers (2.0–7.5%) and in non-fasting period (6.7%) compared with their counterparts. This finding suggests that activities which can improve the child feeding practices should include improving maternal feeding behavior, as many scholars evidenced their association, including ours [9,21,48].

### 4.2. Nutritional Status of 6–23-month-Old Children

In the present study, the prevalence of wasting, underweight and stunting in the 6–23 months old children was between 4.4–4.8%, 11.7–15.7% and 31.6–33.7%, respectively. These results are lower than the findings for the Tigray region and national figures in last two DHS studies in Ethiopia. For example, wasting, underweight, and stunting in under-five aged children were 10.3% and 9.7%, 35.1% and 28.7%, and 51.4% and 44.4% in DHS 2011 (Tigray region and national, respectively). Except for wasting, these figures further decreased to 23.0% and 23.6% for underweight, and 39.3% and 38.4% for stunting in the DHS 2016 study [28,29]. This could indicate an improvement in child nutritional status, both regionally, and at the national level.

In this study, the median WAZ of children of fasting mothers was lower compared to children of non-fasting mothers, both in fasting and non-fasting periods. The WAZ of children from non-fasting mothers’ sub-group also improved during the non-fasting period. Similarly, the prevalence of underweight children from the fasting sub-group was higher than that of children from the non-fasting sub-group, in both periods. Likewise, the mean standard deviations (SD) of WAZ, WHZ and HAZ of children from the fasting mothers sub-group were lower compared to children from non-fasting mothers during the fasting period. Similarly, the proportion of wasted children was higher in the fasting sub-group compared to those children from non-fasting mothers during the fasting period. Conversely, relatively more children from non-fasting mothers were stunted than from fasting mothers. This could be due to the higher number of older children in the non-fasting group. Similar findings were observed in studies conducted in the Tigray region, northern Ethiopia [4]. Multivariate logistic regression showed that children of mothers who fasted during the pregnancy and lactation period were more likely to be undernourished (stunting, underweight, and wasting) than those children of mothers who were not fasting in the same periods. These indicate that, maternal fasting during pregnancy, and lactation not only affect the nutritional status of the mother themselves [9], but also their fetus/breastfed child. To increase the awareness on the effect of fasting practices on the undernutrition, the Ethiopian Orthodox Church (EOTC) with the United States Agency for International Development (USAID) multi-sectoral nutrition project, Empowering New Generations to Improve Nutrition and Economic opportunities (ENGINE) held a consultative meeting with religious leaders and church scholars to clarify fasting practices for children, pregnant and lactating women in line with religious guidelines. This was done after a sermon guide was developed in 2016, based on the church teachings and outlining the laws and regulations related with fasting and nutrition. According to the joint press release by EOTC and USAID/ENGINE, the sermon guide highlighted the exemption of mothers and their babies from fasting during the first 1000 days [49]. The sermon guide also emphasized the cooking utensils to be used for complementary food preparation during fasting seasons, as many mothers were not happy to prepare complementary food from the animal sources during fasting seasons due to fear of contamination of family food [10]. Therefore, strengthening the activities started by the Church and the involvement of religious institutions and leaders in the existing national multi-sectoral approach activities would accelerate and sustain the reduction of nutrition-related problems in the country.

Previous studies in Africa and Southeast Asian countries demonstrated that children in the age between 13–23 months were more associated with underweight and stunting compared to those who were aged 6–12 months [28,29,50,51,52,53,54,55,56,57,58,59,60,61]. Similar findings were also revealed in our study. This could be related to feeding practices, contamination and poor quality complementary food, which is mainly cereal based [4]. Similarly, maternal illiteracy was found to be a predictor variable for underweight and wasting. The former result is consistent with previous studies in Ethiopia and other low and middle income countries [28,29,62,63,64,65]. Similarly, findings in Ethiopian DHS also showed that childhood wasting was associated with maternal illiteracy [28,29]. This indicates that educating mothers will be an important activity for improving the nutritional status of children in a given community. Likewise, compared to children whose mothers were housewives, children of farmer mothers were more exposed to being underweight. This might be related to less time allocation for child care due to a high work load and absence from the home. Farm work also expends more energy, and mothers may be more frequently exhausted, which could result in poor appetite and might affect the quality and quantity of breast milk. Moreover, children who did not take the first milk (colostrum) after their birth were more stunted than those who did. This could be related to infection emanating from pre-lacteal foods, which is a predictor for child stunting in North-west Ethiopia [58]. Therefore, activities which improve the colostrum intake (e.g., -antenatal care) should be done in the study area [66]. Furthermore, children who were living in households without toilets were more exposed to stunting compared to those from households owning toilets. This could be related to poverty in general, a well-known factor for malnutrition. Additionally, contamination of the soil due to open defecation increases the risk for frequent episodes of childhood diarrhea, which has a negative impact on the nutritional status of sub-Saharan African children [50].

However, this study has the following limitations: This study considered only the lent fasting period, among the seven official fasting periods in Ethiopian Orthodox religion. It also included only children aged between 6–23 months, out of those children below seven years of age, who are exempted from the official fasting in the religion.

## 5. Conclusions

This study showed that there is high prevalence of stunting (31.6–33.7%), underweight (11.7–15.7%), and wasting (4.4–4.8%) in the study area; however, these values are lower than those reported earlier in the Tigray region (39.3%, 23.0% and 11.1%), and of national figures (38.4%, 23.6% and 9.9%). These are indications of improvement of child nutrition in rural Tigray. However, the prevalence’s are still higher, both in children of fasting mothers and the study population especially during Ethiopian Orthodox lent fasting period. Thus, religious fasting should not go against the existing nutritional intervention modalities. Mother’s fasting during the lactation period of an indexed child was the common predictor for child stunting, underweight and wasting. Maternal illiteracy and age of children and maternal fasting during pregnancy of indexed children were also common variables affecting combined effects of underweight and wasting, and underweight and stunting, whereas, the type of job of mothers and lack of colostrum feeding of children after birth were associated with underweight and absence of a toilet in the household with the stunting of children. Hence, improving maternal education and reducing the work burden in farming activities or promotion and support of mothers in off-farm income generation nearby their households are recommended. Improving household latrine coverage and breastfeeding of colostrum should be encouraged and strengthened.

‘Grains, roots, and tubers’ was the most frequently eaten food group by 6–23-month-old children, followed by ‘legumes and nuts’ and then ‘other fruits and vegetables’. Therefore, to increase nutrients and improve the palatability of grains and legumes, traditional food processing techniques like dehulling/dehusking, soaking and germination, and slightly roasting are crucial. Additionally, the proportion of children who consumed dairy products, flesh foods and eggs who met the MDD was small. The average DDS of the children was also low, being worst in children of fasting mothers especially during the lent fasting period. Thus, regardless of the fasting period, diet diversity should be properly addressed in preparation of complementary foods by incorporating animal products (dairy products; flesh foods; eggs), vitamin A-rich fruits and vegetables. In general, nutritional status is affected by maternal fasting status during pregnancy and lactation periods, and during the lent fasting period in rural Tigray, Ethiopia. Therefore, without strengthening and involvement of religious institutions and leaders in the existing nutrition improvement activity by the Church and in the National Nutrition Program (NNP) of, in particular nursing mothers and growing children, reduction of undernutrition would not be successful and sustainable. As this study is focused on the food consumption and growth index pattern of 6–23-month-old children, further studies on physiological consequences of levels of micronutrients or bio-markers are recommended.

## Figures and Tables

**Table 1:** Socio-demographic and economic characteristics of the study participants (*n* = 567) in rural Tigray, Northern Ethiopia (February–June, 2017).

Characteristics (*n* = 567)		Number	Percent
Religion of mother	Orthodox	565	99.6
	Other	2	0.4
Mother fasting during pregnancy period of indexed child	Yes	156	27.5
Mother fasting during lactation period of indexed child	Yes	176	31.0
Mother’s education	Literate	369	65.1
Mother’s occupation	Housewives	449	79.2
	Farmer	80	14.1
	Other	38	6.7
Family size	≤5	218	38.4
Household food security status	Food secure	166	29.3
Household toilet presence	Yes	455	80.2

**Table 2:** Basic information, anthropometric status and consumption pattern of 6–23-month-old children in lent fasting and non-fasting periods in rural Tigray, Ethiopia (February–June, 2017).

		Lent Fasting Period (*n* = 567)			Non-Fasting Period (*n* = 505)		
		Children of Non-Fasting Mothers	Children of Fasting Mothers	Total	Children of Non-Fasting Mothers	Children of Fasting Mothers	Total
		*N* (%)	*N* (%)	*N* (%)	*N* (%)	*N* (%)	*N* (%)
Age of child	≤12 months	168 (29.6)	71 (12.5)	239 (42.2)	103 (20.4)	45 (8.9)	148 (29.3)
	13–23 months	223 (39.3)	105 (18.5)	328 (57.8)	246 (48.7)	111 (22.0)	357 (70.7)
Sex of child	Male	215 (37.9)	104 (18.3)	319 (56.3)	190 (37.6)	93 (18.4)	283 (56.0)
	Female	176 (31.0)	72 (12.7)	248 (43.7)	159 (31.5)	63(12.5)	222 (44.0)
Colostrum intake after birth	Yes	381 (67.2)	169 (29.8)	550 (97.0)			
Stunted child		104 (18.3)	75 (13.2)	179 (31.6)	110 (21.8)	60 (11.9)	170 (33.7)
Underweight child		42 (7.4)	47(8.3)	89(15.7)	27 (5.3)	32 (6.3)	59 (11.7)
Wasted child		7 (1.2)	20 (3.5)	27 (4.8)	10 (2.0)	12 (2.4)	22 (4.4)
Grains, roots and tubers consumption		357 (63.0)	164 (28.9)	521 (91.9)	339 (67.1)	147 (29.1)	486 (96.2)
Legumes and nuts consumption		257 (45.3)	112 (19.8)	369 (65.1)	250 (49.5)	108 (21.4)	358 (70.9)
Other fruits and vegetables consumption		104 (18.3)	49 (8.6)	153 (27.0)	138 (27.3)	52 (10.3)	190 (37.6)
Dairy products consumption		33 (5.8)	15 (2.6)	48 (8.5)	41 (8.1)	9 (1.8)	50 (9.9)
Flesh foods consumption		0 (0.0)	3 (0.5)	3 (0.5)	4 (0.8)	4 (0.8)	8 (1.6)
Eggs consumption		71 (12.5)	34 (6.0)	105 (18.5)	87 (17.2)	38 (7.5)	125 (24.8)
Vitamin A-rich fruits and vegetables consumption		0 (0.0)	5 (0.9)	5 (0.9)	4 (0.8)	2 (0.4)	6 (1.2)
MAD		10 (1.8)	3 (0.5)	13 (2.3)	25 (5.0)	9 (1.8)	34 (6.7)
MDD		9 (1.6)	5 (0.9)	14 (2.5)	25 (5.0)	10 (2.0)	35 (6.9)
MMF		291 (51.3)	130 (22.9)	421 (74.3)	258 (51.1)	121 (24.0)	379 (75.0)

MAD = minimum acceptable diet; MDD = minimum diet diversity; MMF = minimum meal frequency; Fasting: Abstaining from eating any animal source foods during the seven official fasting periods of the Ethiopian Orthodox Tewahedo Church with/without abstaining from any foods and water until 12:00 noon and beyond of the fasting days; Fasting mother: a mother who usually abstained from eating any animal source foods (flesh foods, eggs and dairy products) during the official fasting periods of the Ethiopian Orthodox Tewahedo Church with/without abstaining from any foods and water until 12:00 noon and beyond of the fasting days, after the baptism day (40 and 80 days for a baby boy and girl, respectively) until the breast feeding indexed child was aged 23 months; Children of fasting mothers: Those children who were born of fasting mothers; Children of non-fasting mothers: Those children who were born of mothers who were not practicing fasting.

**Table 3:** Multivariate logistic regression: associated factors of malnutrition of 6–23-month-old children regarding underweight, stunting and wasting in rural Tigray, Ethiopia (*N* = 567).

Variables	Predicting Category	OR	95% CI	*p*-Value
Underweight				
Age of child	13–23 months	1.79	1.07, 3.00	0.028
Mother fasting during pregnancy period of indexed child	Not fasting	2.12	1.29, 3.51	0.003
Mother fasting during lactation period of indexed child	Not fasting	2.57	1.56, 4.23	0.000
Mother’s education	Illiterate	1.79	1.10, 2.90	0.019
Mother’s occupation	Farmer	2.28	1.25, 4.16	0.021
Hosmer-Lemeshow test: *p* = 0.92, C-statistic: AUC = 0.73; 95% CI 0.68–0.79				
Stunting				
Age of child	13–23 months	3.07	2.04, 4.63	0.000
Child colostrum intake status	No colostrum after birth	4.10	1.42, 11.82	0.009
Mother fasting during pregnancy period of indexed child	Not fasting	1.83	1.21, 2.77	0.005
Mother fasting during lactation period of indexed child	Not fasting	1.81	1.20, 2.72	0.005
Toilet presence	No toilet in the household	1.67	1.06, 2.64	0.026
Hosmer-Lemeshow test: *p* = 0.98, C-statistic: AUC = 0.70; 95% CI 0.66–0.75				
Wasting				
Mother fasting during lactation period of indexed child	Not fasting	6.78	2.78, 16.40	0.000
Mother education	Illiterate	2.24	1.01, 4.98	0.047
Hosmer-Lemeshow test: *p* = 0.47, C-statistic: AUC = 0.78; 95% CI 0.70–0.86				

AUC = Area under the curve; OR = Odd ratio; CI: Confidence interval.

**Table 4:** Food consumption pattern of 6–23-month-old children of fasting and non-fasting mothers during lent fasting and non-fasting periods in rural Tigray, Northern Ethiopia.

Parameters	Children of Fasting Mothers (*n* = 156)			Children of Non-Fasting Mothers (*n* = 349)		
	Lent Fasting Period	Non-Fasting Period	Significance	Lent Fasting Period	Non-fasting Period	Significance
	*N* (%)	*N* (%)	*p*-Value	*N* (%)	*N* (%)	*p*-Value
Grains, roots and tubers consumption	145 (92.9)	147 (94.2)	0.815 ^ns^	317 (90.8)	339 (97.1)	0.000 *
Pulses and nuts consumption	97 (62.2)	108 (69.2)	0.228 ^ns^	228 (65.3)	250 (71.6)	0.047 *
Other fruits and vegetables	45 (28.8)	52 (33.3)	0.427 ^ns^	91 (26.1)	138 (39.5)	0.000 *
Dairy products consumption	14 (9.0)	9 (5.8)	0.332 ^ns^	31 (8.9)	41 (11.7)	0.203 ^ns^
Flesh food consumption	3 (1.9)	4 (2.6)	1.000 ^ns^	0 (0.0)	4 (1.1)	NA
Eggs consumption	30 (19.2)	38 (24.4)	0.302 ^ns^	64 (18.3)	87 (24.9)	0.017 *
Vitamin A fruits and vegetables	5 (3.2)	2 (1.3)	0.453 ^ns^	0 (0.0)	4 (1.4)	NA
MAD	3 (1.9)	9 (5.8)	0.146 ^ns^	7 (2.0)	25 (7.5)	0.001 *
MDD	5 (3.2)	10 (6.4)	0.302 ^ns^	6 (1.7)	25 (7.2)	0.001 *
MMF	114 (73.1)	121 (77.6)	0.349 ^ns^	256 (73.4)	258 (73.9)	0.927 ^ns^

Data analysis using McNemar’s test, significant level at *p* < 0.05; NA = the data were not appropriate for analysis; MAD: minimum acceptable diet, MDD = minimum diet diversity, MMF: minimum meal frequency; ns- not significantly different at *p* < 0.05; * = significantly different at *p* < 0.05.

**Table 5:** Anthropometric measurements (WAZ, WHZ and HAZ) of 6–23-month-old children of fasting and non-fasting mothers in the lent fasting period in rural Tigray, Ethiopia.

Variables	Fasting Mother (*N* = 176)	Non-Fasting Mother (*N* = 391)	*p*-Value
	Mean (Standard Deviation)	Mean (Standard Deviation)	
WAZ	−1.25 (1.15)	0.77 (1.09)	0.000 *
WHZ	−0.58 (1.15)	−0.19 (1.06)	0.000 *
HAZ	−1.60 (1.35)	−1.24 (1.34)	0.003 *

HAZ = height for age; WHZ = weight for height; WAZ = weight for age; data analysis using independent sample *t*-test, significance level declared at *p* < 0.05; * = significantly different at *p* < 0.05.

**Table 6:** Comparison of 6–23-month-old children’s WAZ and DDS values between lent fasting and non-fasting periods in rural Tigray, Ethiopia.

Variables	Data Collection Period						
	Lent Fasting			Non-Fasting			
	Mean (SD)	Median (IQR)	Range (Min, Max)	Mean (SD)	Median (IQR)	Range (Max, Min)	Sign
Children WAZ of fasting mother	−1.25 (1.14)	−1.22 (−2.03, −0.54)	−4.33, 2.52	−1.16 (1.14)	−1.11 (−1.83, −0.46)	−4.37, 3.83	0.034 *
Children WAZ of non-fasting mother	−0.74 (1.08)	−0.79 (−1.48, −0.07)	−4.97, 2.70	−0.66 (1.02)	−0.69 (−1.32, −0.11)	−0.5.08, 2.32	0.153 ^ns^
Children DDS of fasting mother	2.09 (1.02)	2.00 (1.50, 2.50)	0.00, 7.00	2.43 (0.93)	2.50 (2.00, 3.00)	0.00, 4.5)	0.000 *
Children DDS of non-fasting mother	2.04 (0.88)	2.00 (1.50, 2.50)	0.00, 5.00	2.50 (0.95)	2.50 (2.00, 3.00)	0.00, 7.00	0.000 *

Fasting mothers (*n* = 156); non-fasting mother (*n* = 349); data analyses using the Wilcoxon signed-rank test, significant level at *p* < 0.05; WAZ = weight for age; DDS = diet diversity score; ns- not significantly different at *p* < 0.05; * = significantly different at *p* < 0.05.
